# Qualitative assessment of EOB-GD-DTPA and Gd-BT-DO3A MR contrast studies in HCC patients and colorectal liver metastases

**DOI:** 10.1186/s13027-019-0264-3

**Published:** 2019-11-27

**Authors:** Vincenza Granata, Roberta Fusco, Francesca Maio, Antonio Avallone, Guglielmo Nasti, Raffaele Palaia, Vittorio Albino, Roberto Grassi, Francesco Izzo, Antonella Petrillo

**Affiliations:** 1 Division of Radiology, ISTITUTO NAZIONALE TUMORI – IRCCS - FONDAZIONE G. PASCALE, NAPOLI, ITALIA, Naples, Italy; 2 Division of Radiology, Università degli Studi di Napoli Federico II, Naples, Italy; 3 Division of Abdominal Oncology, ISTITUTO NAZIONALE TUMORI – IRCCS - FONDAZIONE G. PASCALE, NAPOLI, ITALIA, Naples, Italy; 4 Division of Abdominal Surgical Oncology, Hepatobiliary, ISTITUTO NAZIONALE TUMORI – IRCCS - FONDAZIONE G. PASCALE, NAPOLI, ITALIA, Naples, Italy; 5 Division of Radiology, Università degli Studi della Campania Luigi Vanvitelli, Naples, Italy

**Keywords:** HCC, Colorectal liver metastases, Magnetic resonance imaging, EOB-GD-DTPA, Gd-BT-DO3A

## Abstract

**Aim:**

To compare liver-specific EOB-GD-DTPA and liver-non-specific Gd-BT-DO3A MR, in hepatocellular carcinoma (HCC) and liver colorectal metastases.

**Material and methods:**

Seventy HCC patients with 158 nodules and 90 colorectal liver metastases (mCRC) with 370 lesions were included in the retrospective analysis. HCC patients underwent MR at 0 time (MR0), after 3 (MR3) and 6 months (MR6) using two different CM; 69 mCRC patients underwent MR with Gd-EOB-BTPA and 21 mCRC patients with Gd-BT-DO3A. We evaluated arterial phase hyperenhancement, lesion-to-liver contrast during portal phase, hepatobiliary phase parenchymal hyperenhancement.

**Results:**

In HCC patients arterial phase hyperenhancement degree was statistically higher (*p* = 0.03) with Gd-BT-DO3A (mean 4) than GD-EOB-DTPA (mean 2.6), while we found no significant statistical differences among mean (2.6) values at MR0 and MR6 using GD-EOB-DTPA. For all 209 patients underwent Gd-EOB-DTPA, we found that lesion-to-liver contrast during portal phase mean value was 4 while for patients underwent MR with Gd-BT-DO3A was 3 (*p* = 0.04). For HCC hepatobiliary phase parenchymal hyperenhancement mean value was 2.4. For mCRC patients: among 63 patients underwent previous chemotherapy hepatobiliary phase parenchymal hyperenhancement mean value was 3.1 while for 6 patients no underwent previous chemotherapy was 4 (*p* = 0.05).

**Conclusions:**

Gd-EOB-DTPA should be chosen in pre surgical setting in patients with colorectal liver metastases.

## Introduction

Magnetic resonance imaging (MRI) is the gold standard in the detection and characterization of focal and diffuse liver diseases [1, 2], providing morphological and functional data by Diffusion Weighted imaging (DWI) and Dynamic Contrast Enhanced (DCE) sequences [3]. Contrast medium rises the detection rate of focal liver lesions by increasing the lesion-to-liver contrast. Thanks to its pharmacokinetics, as described in detail previously, “the contrast agent may be increase the characterization of lesions by assessing changes in the perfusion, endothelial permeability, extracellular diffusion, hepatocytic uptake and biliary excretion, relating to the transfer rates between extracellular and intracellular spaces” [1]. MR contrast media (CM) are characterized into non-specific CM that distribute into the vascular and extravascular extracellular spaces, and liver-specific CM, taken up by liver cells [4]. In the clinical practice, the most used CM are gadobenate dimeglumine (Gd-BOPTA; MultiHance, Bracco Imaging, Milan, Italy), and gadolinium ethoxybenzyl diethylenetriamine pentaacetic acid (Gd-EOB-DTPA; Primovist, Bayer-Schering Pharma, Berlin, Germany) [4]. Gd-EOB-DTPA and Gd-BOPTA offer data about lesion vascularity, by analyzing their pharmacokinetics, in the different phases of contrast study, and functional data in the hepatobiliary phase performed either 20 min (Gd-EOB-DTPA) or 60–120 min (Gd-BOPTA) after injection. With Gd-EOB-DTPA, almost 50% of the injected dose is taken up by hepatocytes and excreted into the bile, while with the Gd-BOPTA only the 5% of CM is taken up by hepatocytes and excreted into the bile [1]. During the hepatobiliary phase, normal liver parenchyma is uniformly hyperintense. If there are hepatic structural changes, hepatobiliary phase is weakened or absent: primitive or secondary liver lesions are not hyperintense during this phase of contrast study since missing normal hepatocytes. So hepatobiliary phase offers data about the structure and function of liver [1–5]. Despite the proven advantages of hepatospecific contrast agents, recent studies showed that suboptimal image quality is frequently observed in the arterial phase imaging with Gd-EOB-DTPA [6–9] that could have negative effects on the characterization of hepatic lesions.

Aim of this study is to compare liver-specific EOB-GD-DTPA and liver-non-specific Gadobutrol (Gd-BT-DO3A) CM, in the detection and characterization of hepatocellular carcinoma (HCC) and liver colorectal metastases (mCRC), evaluating the advantages and limits of each one.

## Materials and methods

### Patient population

A retrospective study, approved by Local Ethical Committee, was performed through a computerized search of medical records on patients underwent liver MR imaging for HCC and liver metastases from May 2010 to May 2018. All data were collect with the maximal preservation of patients’ privacy. For HCC populations, inclusion criteria were subjects with liver focal lesions with diameter between 1 to 2 cm with no “typical HCC” according to major and ancillary imaging features of LI-RADS [2, 3]; subjects that underwent MR study at time 0, after 3 and after 6 months according to our study protocol; all MR sequences must be considered diagnostic by expert radiologist to be included in the analysis. For mCRC patients inclusion criteria were subjects that underwent MR studies to assess the resectability or to assess perfusion parameters of the lesions; the high quality of MR images in order to assess all detected lesions.

For all patients, exclusion criteria were final imaging report did not confirm the HCC or colorectal metastases and low quality MR images.

After reviewing the medical records, we found 274 patients that met inclusion criteria, however 90 patients were excluded because the final imaging report was did not confirm the HCC or colorectal metastases and 24 because the quality of all contrast study phases was low.

The final study population included 70 HCC patients (33 women and 37 men, mean age 68 years, range, 52–83 years) with 158 nodules (tumor diameter between 1 to 2 cm) and 90 colorectal liver metastases patients (42 men and 48 women, mean age 63 years, range 38–80 years).

According to our imaging protocol, all HCC patients were subject to MR study at time 0 (MR0), after 3 (MR3) and 6 months (MR6) using two different CM. Gd-EOB-BTPA was injected at MR0 and MR6, while Gd-BT-DO3A was injected at MR3. The mean interval between pathologic examination and last MR study (MR6) was 15 days (range 4–28 days). This protocol is chose for nodules that are not classify as “typical HCC”, according to major and ancillary imaging features of LI-RADS [2, 3]. No patients were subject any treatment between T0 and T6. In this study we evaluated few patients (25 out of 70, 35.7%) that we assessed in our previous study [2].

Among colorectal liver metastases patients, in 47 subjects the primary cancer was located in the rectum and in the remaining 43 subjects it was located in the colon. All patients had an adenocarcinoma and 63 patients underwent previous chemotherapy. An overall number of 370 lesions were counted in 90 patients (mean 6.4/patient, range 1–31).

Sixty-nine patients (63 patients had chemotherapy and 6 had no history of chemotherapy) underwent MR study with Gd-EOB-BTPA as a pre- surgical study to assess the resectability of the lesions, and 21 underwent MR study with Gd-BT-DO3A since we decided the use of this contrast medium for perfusion lesion assessment.

The diagnosis of metastases was retrospectively established on the basis of surgery in 42 cases, of MR follow-up in 29 cases, and by other imaging modalities (multidetector computed tomography (MDCT) and contrast-enhanced ultrasound (CEUS) in agreement) in 19 cases. The mean interval between pathologic examination and MR study was 9 days (range 4–18 days).

### MR imaging protocol

MR studies were performed using a 1.5 T MR (Magnetom Symphony, with Total Imaging Matrix Package, Siemens, Erlangen, Germany) with 8-element body and phased array coils. The MRI examination consisted of basal images taken before IV administration of contrast medium and then functional dynamic sequences obtained after IV injection of CM, acquiring the last series of images, when we used hepatospecific CM, with a delay of 20 min during the hepatobiliary excretion of the CM. The baseline sequences obtained before IV contrast medium were coronal TRUFISP T2-weighted free breathing; axial Half-Fourier Acquisition Single-Shot Turbo Spin-Echo (HASTE) T2-weighted, with controlled respiration, without and with fat-suppressed (FS) gradient-echo pulse; coronal HASTE T2-weighted, without FS; axial flash in-out phase T1-weighted, with controlled respiration; Volumetric Interpolated Breath-hold Examination (VIBE) T1-weighted SPAIR with controlled respiration**;** diffusion weighted imaging (DWI) with planar echo-pulse sequence (EPI) at several b value *b* value 0, 50, 100, 200, 400, 600, and 800 s/mm^2^. As liver-specific CM, the EOB-Gd-BPTA (Primovist, Bayer Schering Pharma, Germany) was employed. All patients received 0.1 ml/kg of Gd-EOB-BPTA by means of a power injector (Spectris Solaris® EP MR, MEDRAD Inc., Indianola, IA, USA), at an infusion rate of 1 ml/s. As non-specific agent the Gd-BT-DO3A (Gadovist, Bayer Schering Pharma, Germany) was employed. All patients received 0.1 ml/kg of Gd-BT-DO3A by means of a power injector (Spectris Solaris® EP MR, MEDRAD Inc., Indianola, IA, USA), at an infusion rate of 2 ml/s. After contrast medium administration, VIBE T1-weighted FS (SPAIR) sequences were acquired in different phases: hepatic arterial (35 s delay), portal venous (90 s), equilibrium or transitional (120 s) phases correlated to CM employed, and hepatobiliary excretion (20 min) phase post Gd- EOB-BPTA. Details of sequence parameters were reported in Table 1.

**Table 1:** Pulse Sequence Parameters on MR studies

Sequence	Orientation	TR/TE/FA (ms/ms/deg.)	AT (min.)	Acquisition Matrix	Slice thickness/Gap (mm)	Fat Suppression
TRUFISP T2-W	Coronal	4.30/2.15/80	0.46	512 × 512	4 / 0	without
HASTE T2-W	Axial	1500/90/170	0.36	320 × 320	5 / 0	Without and with (SPAIR)
HASTE T2w	Coronal	1500/92/170	0.38	320 × 320	5 / 0	without
In-Out phase T1-W	Axial	160/2.35/70	0.33	256 × 192	5 / 0	without
DWI	Axial	7500/91/90	7	192 × 192	3 / 0	without
VIBE T1-W	Axial	4.80/1.76/12	0.18	320 × 260	3 / 0	with (SPAIR)

Note**.** *W* Weighted, *TR* Repetition time, *TE* Echo time, *FA* Flip angle, *AT* Acquisition time, *SPAIR* Spectral Adiabatic Inversion Recovery, *HASTE* Half-Fourier acquisition single-shot turbo spin-echo, *DWI* Diffusion-weighted imaging *VIBE* Volumetric interpolated breath hold examination

### Images analysis

Three hepatic radiologists with more of 15 years of experience, retrospectively and independently reviewed all images. A consensus evaluation was performed when there was disagreement between the readers. The observers were blinded to clinical history and previous imaging studies.

The radiologists assessed all detected lesions in all sequences; the diagnosis of HCC nodules was based on LIRADS major features [2, 3]. A lesion was considered metastases on the basis on the MRI features [5]. The gold standard was pathologic examination on surgical specimen.

For HCC patients each observer independently evaluated the presence of arterial phase hyperenhancement using a four-point scale (1 = absent, 2 = low intensity, 3 = mild intensity, 4 = high intensity), to compare the efficacy of the two different contrast media and to evaluate hyperenhancement.

For all patients each observer independently evaluated the lesion-to-liver contrast during portal phase of contrast study using a four-point scale (1 = absent or minimal, 2 = mild, 3 = moderate, 4 = high), to compare the efficacy of the two different contrast media in assessment of metastases, and, for HCC patients, in assessment of wash-out.

For all patients each observer independently evaluated the presence of hepatobiliary phase parenchymal hyperenhancement using a four-point scale (1 = absent, 2 = low intensity, 3 = mild intensity, 4 = high intensity), to evaluate the efficacy of EOB-GD-DTPA as a tool to assess the hepatic functionality.

Also, for all patients, each observer independently evaluated the degree of image quality degradation caused by respiratory ghost, pulsatile blood flow ghost, and susceptibility artifacts using a four-point scale (1 = absent or minimal, 2 = mild, 3 = moderate, 4 = severe) for arterial phases of studies. A “severe” score indicated that an image was uninterpretable and a “mild” score indicated that the artifacts did not affect interpretation.

### Statistical analysis

Data were expressed in terms of mean value ± range. Mann Whitney and Kruskal Wallis non-parametric test were performed to emphasize significant statistically difference between mean values in different population subgroups. A *p* value < 0.05 was considered statistically significant.

All analyses were performed using Statistics Toolbox of Matlab R2007a (The Math-Works Inc., Natick, MA).

## Results

**Fig. 1:**
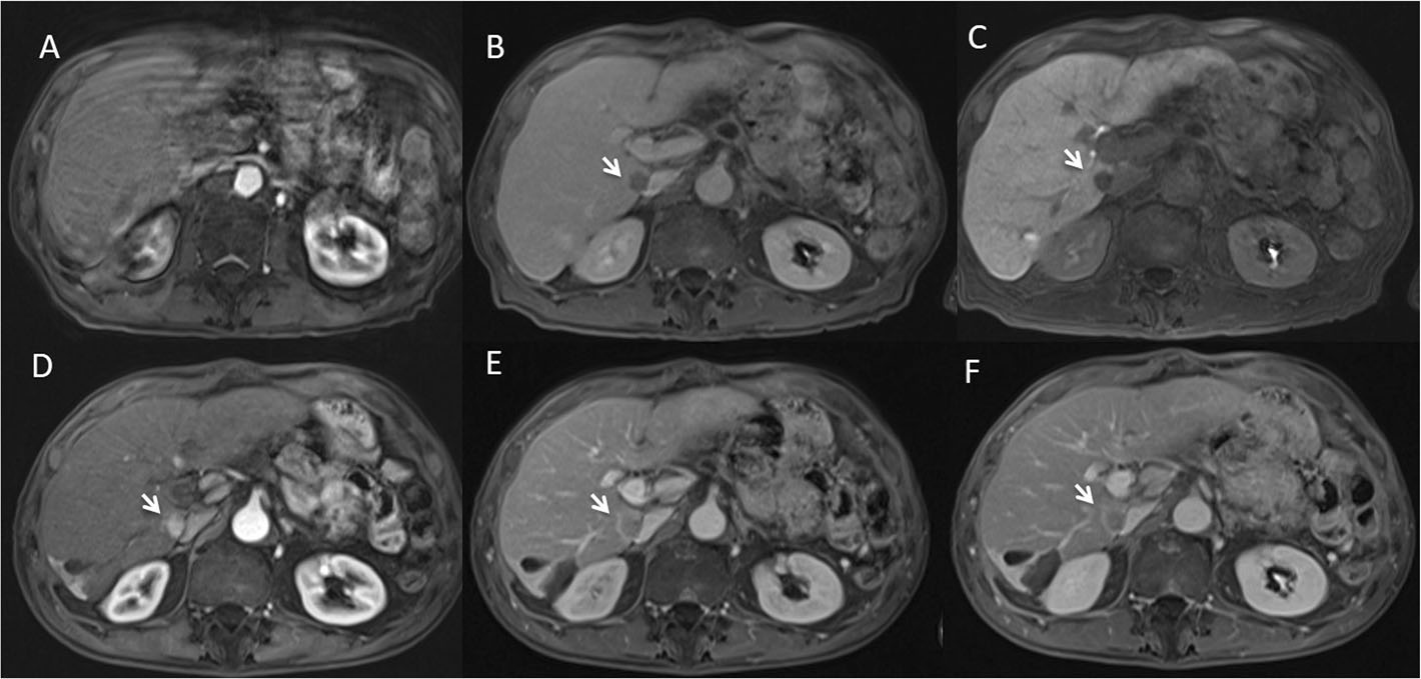
Man 73 years with HCC nodule. In **a**, **b** and **c** GD-EOB-DTPA M0 study. In **a** arterial phase (VIBE T1-W FS), the HCC located to VII segment is not detect and it is due to lower quality of this phase. In **b** (late phase of contrast study) and **c** (HPB phase of contrast study) the nodule is detected (arrows). In D, E and F Gd-BT-DO3A M3 study. In **d** (arterial phase) the HCC nodule (arrow) shows wash-in with wash-out and capsule appearance during portal (**e**) and equilibrium (**f**) phase of contrast study

In HCC patients the degree of hyperenhancement of arterial phase was statistically higher (p value = 0.032 at Mann Whitney test) with Gd-BT-DO3A (mean value 4) than GD-EOB-DTPA (mean value 2.6), while we found no significant statistical differences among mean (2.6) values at MR0 and MR6 using GD-EOB-DTPA in HCC patients (*p* value > 0.05 at Mann Whitney test) (Fig. 1).

**Fig. 2:**
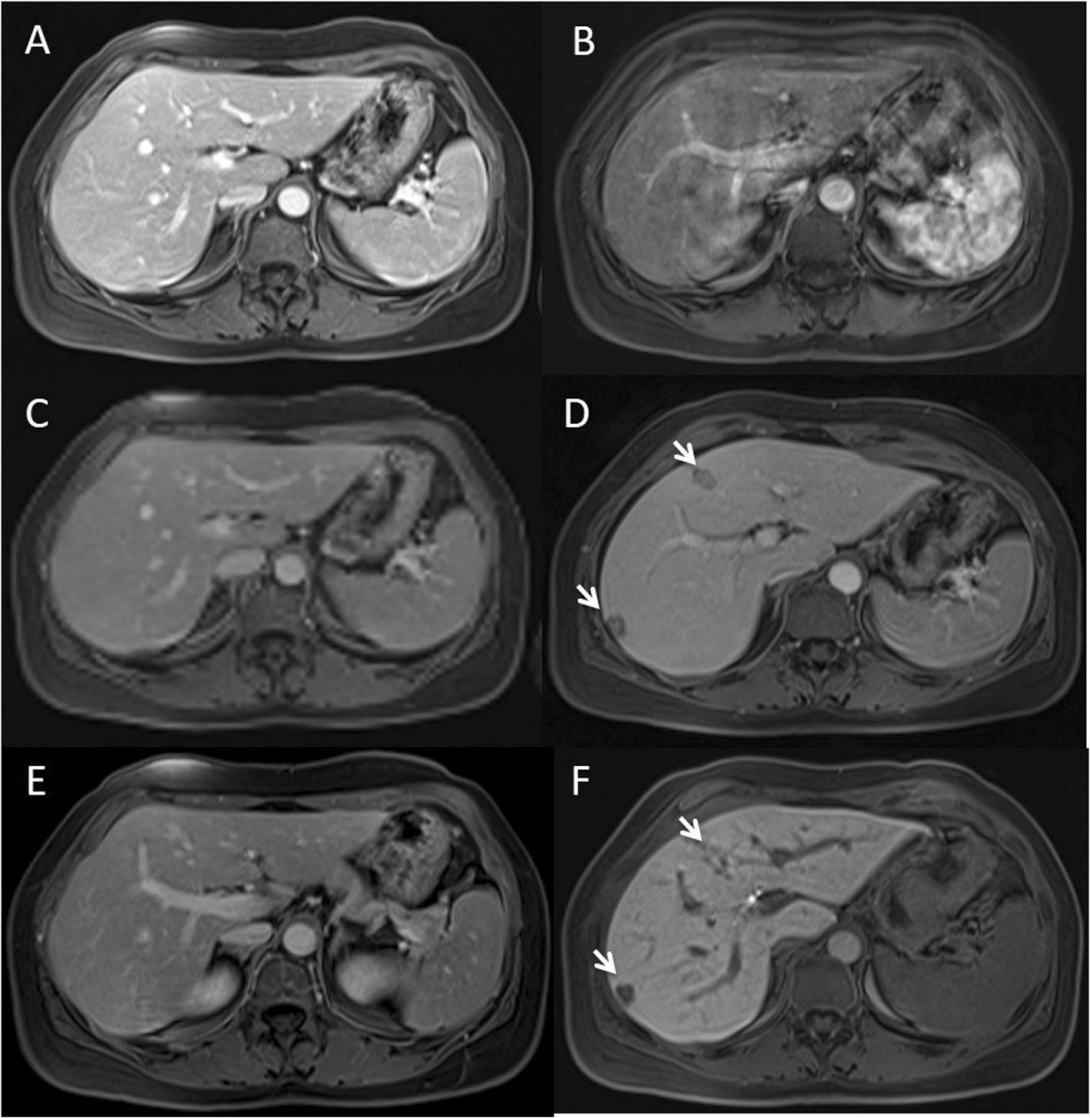
Woman 42 years with colon cancer. Gd-BT-DO3A (**a**, **c** and **e**) and GD-EOB-DTPA (**b**, **d** and **f**) studies performed 15 days away. During Gd-BT-DO3A (**a**, **c** and **e**) contrast study the metastases are nor detected while after GD-EOB-DTPA (**b**, **d** and **f**) contrast agent the metastases are detected (arrow)

For all 209 patients that underwent Gd-EOB-DTPA, we found that the mean value of the lesion-to-liver contrast during portal phase was 4, while for the patients (*n* = 91) that underwent MR study with Gd-BT-DO3A, the lesion-to-liver contrast during portal phase was 3 (range 2–4; the results were statistically significant, *p* value = 0.041 at Mann Whitney test), with lower values in patients that underwent chemotherapy (Fig. 2).

For HCC patients (140 studies evaluated) the mean value of hepatobiliary phase parenchymal hyperenhancement was 2.4 (range 2–4).

For colorectal patients (69 studies evaluated): among 63 patients that underwent previous chemotherapy the mean value of hepatobiliary phase parenchymal hyperenhancement was 3.1 (range 2–4) while for the 6 patients that had no previous chemotherapy the value of hepatobiliary phase parenchymal hyperenhancement according to the score was 4 (the results were statistically significant, p value = 0.045 at Mann Whitney test).

We evaluated 300 arterial phases to assess the image quality degradation. The mean score for all arterial phase with Gd-BT-DO3A (91 studies) was 1, while the mean score for all arterial phase with GD-EOB-DTPA (209 studies) was 3 (range 1–4) (the results were statistically significant, p value = 0.039 at Mann Whitney test). In 52 (24.9%) cases the images, during arterial phase with GD-EOB-DTPA, were unfit for diagnosis. Our results are summarized in Tables 2 and 3.

**Table 2:** Results, in terms of mean value, obtained with Gd-BT-DO3A

Description	Score (Mean Value)
Reader 1	Gd-BT-DO3A
Degree of hyperenhancement of arterial phase	4
Lesion-to-liver contrast during portal phase	3
Quality of arterial phases	1
Reader 2	
Degree of hyperenhancement of arterial phase	4
Lesion-to-liver contrast during portal phase	3
Quality of arterial phases	1
Reader 3	
Degree of hyperenhancement of arterial phase	4
Lesion-to-liver contrast during portal phase	2.9
Quality of arterial phases	1

**Table 3:** Results, in terms of mean value, obtained with GD-EOB-DTPA

Description	Score (Mean Value)
Reader 1	GD-EOB-DTPA
Degree of hyperenhancement of arterial phase	2.7
Lesion-to-liver contrast during portal phase	4
Hepatobiliary phase parenchymal hyperenhancement	2,4 for HCC; 2,9 for mCRC with previous chemotherapy; 4 for mCRC without previous chemotherapy
Quality of arterial phases	2.9
Reader 2	
Degree of hyperenhancement of arterial phase	2.5
Lesion-to-liver contrast during portal phase	4
Hepatobiliary phase parenchymal hyperenhancement	2,5 for HCC; 3 for mCRC with previous chemotherapy; 4 for mCRC without previous chemotherapy
Quality of arterial phases	3
Reader 3	
Degree of hyperenhancement of arterial phase	2.6
Lesion-to-liver contrast during portal phase	4
Hepatobiliary phase parenchymal hyperenhancement	2,4 for HCC; 3,3 for mCRC with previous chemotherapy; 4 for mCRC without previous chemotherapy
Quality of arterial phases	3

## Discussion

At the best of our knowledge this study is the first that compare EOB-GD-DTPA and Gd-BT-DO3A CM in the assessment of HCC and colorectal metastases, evaluating the degree of hyperenhancement of arterial phase and the image quality degradation during this phase of contrast study, the lesion-to-liver contrast during portal phase and the hepatobiliary phase parenchymal hyperenhancement. Although some HCC patients of this study have just evaluated in an our previous study [2], in which we assessed the degree of hyperenhancement and the image quality degradation during arterial phase. Now we evaluated a larger sample size compared to our previous study [2], assessed also mCRC patients and others functional parameters as the lesion-to-liver contrast during portal phase and the hepatobiliary phase parenchymal hyperenhancement. These features are all parameters that a radiologist should assess during the characterization, so that we think that it is important know the advantages and the limits of different contrast agents in order to choose the more appropriate according to the clinical question. Tumour characterization is a complex analysis based on the evaluation of the morphological and functional features of the lesion on the various sequences during MR study. Dynamic contrast enhanced MR imaging during the different phases of contrast study has an important role in this process by showing differences of CM spreading between the vascular and extravascular spaces of tumors and liver parenchyma [6]. GD-EOB-DTPA provides similar data compared to non-specific gadolinium during the arterial and portal phases, but enhancement in the equilibrium phase has contribution from hepatic cellular uptake in addition to contrast in the intravascular and extracellular spaces. So, this phase is better called as “late dynamic phase” [6]. However, recent studies showed that suboptimal image quality is frequently observed in the arterial phase imaging with Gd-EOB-DTPA [7–9]. The phenomenon was named “severe respiratory motion artifact”, and the cause of this is unknown and was first described as acute self-limiting dyspnea [8]. Akai et al. evaluated rapid respiratory effect of Gd-EOB-DTPA in an experimental study on mice [10]. As described in detail previously by [10], “the respiratory effect of gadoteridol and gadopentetate dimeglumine to compare with gadoxetate disodium showed that gadoxetate disodium increased the respiratory rate rapidly, and the effect on respiration tended to be larger than gadoteridol and gadopentetate dimeglumine”. Also Davenport et al. evaluated whether acute transient dyspnea and/or arterial phase image degradation occurs more or less often after injection of Gd-EOB-DTPA compared to gadobenate dimeglumine. They showed that more patient complaints of acute transient dyspnea occurred after gadoxetate disodium administration than gadobenate dimeglumine (14% [14 of 99] vs 5% [5 of 99]). There were significantly more severely degraded arterial phase data sets for gadoxetate disodium than for gadobenate dimeglumine [11]. In our study the image quality degradation was lower with Gd-BT-DO3A (mean score was 1) than with GD-EOB-DTPA (mean score was 3). There was significant statistically difference between the quality on arterial phase with Gd-BT-DO3A and the quality on arterial phase with GD-EOB-DTPA and in 25 cases the images, during arterial phase with GD-EOB-DTPA, were uninterpretable. According to previous reports, the incidence of “transient severe motion” in the arterial phase during Gd-EOB-DTPA studies ranges from 4.8 to 18.3% [9, 12–14]. Dyspnea, whether induced by gadoxetic acid or as the result of breathlessness due to long breath-hold time, may disturb breath-holding and degrade arterial phase image quality. Therefore, according to several studies, reducing breath-hold time, during this phase may be crucial in reducing dyspnea and motion artifacts resulting in improved image quality [15]. Yoo et al. assessed whether a short breath-hold technique can improve arterial phase image quality in Gd-EOB-DTPA MRI compared with a conventional long breath-hold technique and also to objectively evaluate if shortening breath-hold time can reduce gadoxetic acid–related respiratory difficulty by evaluating respiratory-related graphs [15]. They concluded that the short breath-hold MR technique, CAIPIRINHA, showed better image quality with less degraded arterial phase and a lower incidence of breath- hold difficulty and gadoxetic acid–related dyspnea than the conventional long breath-hold technique [15].

When we analyzed the degree of hyperenhancement of arterial phase, we found that the degree was higher with Gd-BT-DO3A than GD-EOB-DTPA, with significant statistically difference (*p* value = 0.02 at Kruskal Wallis test). In dynamic vascular imaging, the total amount of gadolinium administered is of crucial importance as the enhancement during the first-pass is directly related to the intrinsic relaxivity of the CM and its dosage, as long as there are no saturation effects. In dynamic vascular imaging, Gd-BT-DO3A may have the advantage of a higher gadolinium dose [16]. Also MRI with liver-specific contrast medium showed less intense vascular and parenchymal enhancement compared to not specific contrast medium with an early parenchymal enhancement after Gd-EOB-DTPA [16]. So, according to our results we think that the choice of CM should evaluate the rule of arterial phase during the step of characterization of a lesion. In fact, in HCC patients, it is known that the hyper-enhancement during arterial phase is a major feature according to LIRADS [2, 3], although this parameter has a lower sensitivity, specificity, positive predictive value, negative predictive value and diagnostic accuracy than hypointensity on hepatospecific phase [2, 3]. However, when we evaluate a cirrhotic patient, we assessing all hepatic parenchyma in which we can found nodules in different phase of evolution or treated nodules. Considering that the ablated area are evaluated according to modified response evaluation criteria in solid tumors (mRECIST) [17], in HCC patient we should obtain the best quality of the arterial phase. So, in this setting we suggest to evaluate the HCC patients alternating these contrast media [2, 3].

In the assessment of lesion-to-liver contrast during portal phase, we showed that in all 209 patients that underwent Gd-EOB-DTPA, the mean value was 4, while for the patients (*n* = 91) that underwent MR study with Gd-BT-DO3A, the lesion-to-liver contrast during portal phase was 3 (range 2–4), with lower values in patients that underwent chemotherapy. The change in signal of liver parenchyma is the basis for an increased liver-lesion contrast and for an increase in detection, characterization and localization [18]. The arterial and portal phase during dynamic studies after Gd-EOB-DTPA were initially assumed to be comparable to arterial and portal phase after extra-cellular contrast agents [19–21]. However, recent studies showed that MRI with liver-specific contrast agents showed less intense vascular and parenchymal enhancement compared to gadobutrol [22, 23]. Schalkx et al. showed that parenchymal enhancement due to hepatocytic uptake of gadoxetate can start as early as in the late arterial phase [23] similar to the results of Reimer et al. [24], Dahlqvist Leinhard et al. [25], Frydrychowicz et al. [26] and Feuerlein et al. [20] suggesting that hepatocytes uptake may start earlier than late phase. Filippone et al. compared two hepatospecific contrast media and showed that in arterial phase, the S/N ratio was comparable for gadoxetic acid and gadobenate dimeglumine, while in the portal-venous and equilibrium phases, the S/N ratio for gadobenate dimeglumine was less than for gadoxetic acid due to an early accumulation phase [27]. In our opinion this earlier uptake during portal phase causes a higher lesion-to-liver contrast in the gadoxetic acid group compared to gadobutrol group and this is more evident in patients that underwent previous chemotherapy [28]. In fact liver steatosis, post chemotherapy, by decreasing the difference in contrast between hepatic parenchyma and lesions, may reduce diagnostic performance of Gd-BT-DO3A MR studies compared to Gd-EOB-DTPA MR studies. So we suggest that in all patients with liver metastases that underwent chemotherapy in pre surgical setting needs to be evaluated with Gd-EOB-DTPA contrast agent to detect and localize all lesions.

Based on our results, to evaluate the efficacy of GD-EOB- DTPA as a tool to assess the hepatic functionality, for HCC patients (140 studies) the mean value of hepatobiliary phase parenchymal hyperenhancement was 2.4 (range 2–4). For colorectal patients: amoung 63 patients that underwent previous chemotherapy the mean value of hepatobiliary phase parenchymal hyperenhancement was 3.1 (range 2–4) while for the 6 patients that had no previous chemotherapy the mean value of hepatobiliary phase parenchymal hyperenhancement was 4. DCE-MRI with hepatospecific contrast agents has been proposed for the assessment of liver function and staging of liver fibrosis [29, 30]. The possibility to assess regional contrast agent uptake may be useful for preoperative quantification of liver function in patients undergoing hepatic surgery. Several studies performed in patients with chronic liver disease have evaluated the relationships between Gd-EOB-DTPA enhancement of the liver parenchyma and the Child-Pugh classification, indocyanine green retention rate clearance, and liver fibrosis [31–38]. MRI-based indices using the signal intensity measured 20 min after Gd-EOB-DTPA injection, relative enhancement of the liver, increase rate of the liver-to-muscle ratio, liver-to-muscle ratio and liver-to-spleen ratio have all been proposed for the evaluation of liver function [31–39]. However, MRI signal intensity is not an absolute value and it may depend on different parameters so that the quantitative comparison of signal intensity between the images before and after contrast enhancement may not be correlated in a straightforward manner.

Some limitations of our study must be considered. First, since this was a retrospective study, there may have been potential selection bias. Second the assessment is made by a qualitative method and the results are served in consensus so we did not evaluate the inter-reader agreement. Third, different radiological units performed Gd-EOB-DTPA MR studies and Gd-BT-DO3A MR studies; however, we made our best effort to use appropriate images with good quality to evaluate all lesions. Fourth, some patients had a pathologic diagnosis of HCCs or metastases based only on biopsy findings; however, the majority of our study patients had undergone surgical resection.

Another limits of this study in related to the non-evaluation of the role of DWI in the HCC and metastasis assessment [39–42]. DWI has been applied to liver imaging as an excellent tool for detection and characterization of focal liver lesions. The assessment of DW images can be done qualitatively and quantitatively, through the apparent diffusion coefficient (ADC) map. The role of DWI and functional parameters extracted by DWI in HCC and liver metastases patient has been evaluated by different studies, showed that the DWI could be used as a helpful diagnostic tool [43–47].

## Conclusion

Dynamic contrast enhanced MR imaging during the different phases of contrast study has an important role in tumor detection, characterization and localization. Despite the advantages due to use of hepato-specific contrast agents these contrast agents has less intense vascular and parenchymal enhancement compared to not specific contrast agents and parenchymal enhancement due to hepatocytic uptake of gadoxetate acid should start as early as in the late arterial phase. Also, the “transient severe motion” in the arterial phase during Gd-EOB-DTPA MR studies, that should degrade arterial phase image quality so as the degree of hyperenhancement of arterial phase higher with Gd-BT-DO3A than GD-EOB-DTPA, may be considered during HCC studies. Conversely, Gd-EOB-DTPA should be chosen in pre surgical setting in patients with colorectal liver metastases.

## Data Availability

All data are included in the manuscript.
